# Contribution of Organ Vasculature in Rat Renal Analysis for Ochratoxin A: Relevance to Toxicology of Nephrotoxins

**DOI:** 10.3390/toxins7041005

**Published:** 2015-03-24

**Authors:** Peter Mantle, Mehmet A. Kilic, Firdevs Mor, Ozlem Ozmen

**Affiliations:** 1 Centre for Environmental Policy, Imperial College London, London SW7 2AZ, UK; 2 Molecular Biology Section, Department of Biology, Science Faculty, Akdeniz University, Antalya 07058, Turkey; E-Mail: mkilic@akdeniz.edu.tr; 3 Department of Pharmacology and Toxicology, Faculty of Veterinary Medicine, Mehmet Akif Ersoy University, Burdur 15030, Turkey; E-Mail: fmor@mehmetakif.edu.tr; 4 Department of Pathology, Faculty of Veterinary Medicine, Mehmet Akif Ersoy University, Burdur 15030, Turkey; E-Mail: ozlemozmen@mehmetakif.edu.tr

**Keywords:** perfusion *in vivo*, ochratoxin A, renal vasculature, ochratoxin accumulation, DNA adducts, aristolochic acid

## Abstract

Assumptions surrounding the kidney as a target for accumulation of ochratoxin A (OTA) are addressed because the contribution of the toxin in blood seems invariably to have been ignored. Adult rats were maintained for several weeks on toxin-contaminated feed. Using standard perfusion techniques, animals were anaesthetised, a blood sample was taken, one kidney was ligated, and the other kidney perfused with physiological saline *in situ* under normal blood pressure. Comparative analysis of OTA in pairs of kidneys showed marked reduction in the perfused organ in the range 37%–98% (mean 75%), demonstrating the general efficiency of perfusion supported also by histology, and implying a major role of blood in the total OTA content of kidney. Translation of OTA values in plasma to whole blood, and its predicted contribution as a 25% vascular compartment in kidney gave values similar to those in non-perfused kidneys. Thus, apparent ‘accumulation’ of OTA in kidney is due to binding to plasma proteins and long half-life in plasma. Attention should be re-focused on whole animal pharmacokinetics during chronic OTA exposure. Similar principles may be applied to DNA-OTA adducts which are now recognised as occurring in blood; application could also extend to other nephrotoxins such as aristolochic acid. Thus, at least, quantitative reassessment in urological tissues seems necessary in attributing adducts specifically as markers of potentially-tumourigenic exposure.

## 1. Introduction

Ochratoxin A (OTA) is a well-known food-borne mycotoxin, for which the rat kidney is a significant experimental target, potentially evident both in the short-term concerning nephropathy and, after chronic exposure, as renal carcinoma. Several toxicity mechanisms may contribute to the former to diffuse cytotoxicity, according to dose. However, although currently unclear, for the latter, the mechanism for the highly-focal tumourigenesis may be more specific. In all cases, access of circulating OTA to renal parenchyma from the vasculature is important for all toxic expressions. This can at least partly be satisfied by the role of kidney as a principal organ of OTA excretion, in which several thousand independent nephrons function in parallel and via which OTA and its metabolites are known to be eliminated.

In blood, most of the circulating OTA is bound to serum albumins so that it is only the small disassociated component of free OTA molecules that are available for excretion, during which access to potential sites of tumourigenesis can occur.

The aim of the present study was to seek quantitative experimental evidence concerning the extent to which OTA concentration measured in whole kidneys of rats exposed chronically to dietary toxin is located within the renal vasculature. To date, this seems not to have been considered, according to the current literature, when commenting on OTA measured in kidney and blood plasma, or in reviews (e.g., [1]). Therefore, the strategy has been to compare the OTA content of kidneys from rats that have been given OTA contaminated feed for several weeks after isolating one kidney and surgically perfusing the other with a physiological solution. Blood plasma taken concurrently provided the measure of circulating OTA concentration. Necessarily, in the present context, there are general assumptions that the two kidneys in a rat have similar histological architecture, have similar arterial supply, and share excretory function in a reasonably equable way. Organ perfusion *in situ* under terminal anaesthesia is an established procedure for replacing vascular content while maintaining normal *in vivo* intravascular pressure, typically, for example, to ensure penetration of histological fixative to maintain lumen conformation within and around nephrons [2] and to avoid fixation artefacts in the brain due to restricted access of fixative [3,4]. For the present purpose, it is also important to minimise compression of cortical capillaries by the elasticity of the renal capsule by allowing blood pressure to decline slowly. Perfusion efficiency may vary for technical reasons, but this can be monitored subsequently by assessing absence of capillary erythrocytes in histological sections.

Elegant three-dimensional imaging of rat kidney by micro-computed tomography has recognised an approximately 25% *v*/*v* vascular component [5,6], a finding that is particularly relevant here to interpreting any relationship between measured OTA amounts in blood plasma and kidney.

## 2. Results

**Figure 1:**
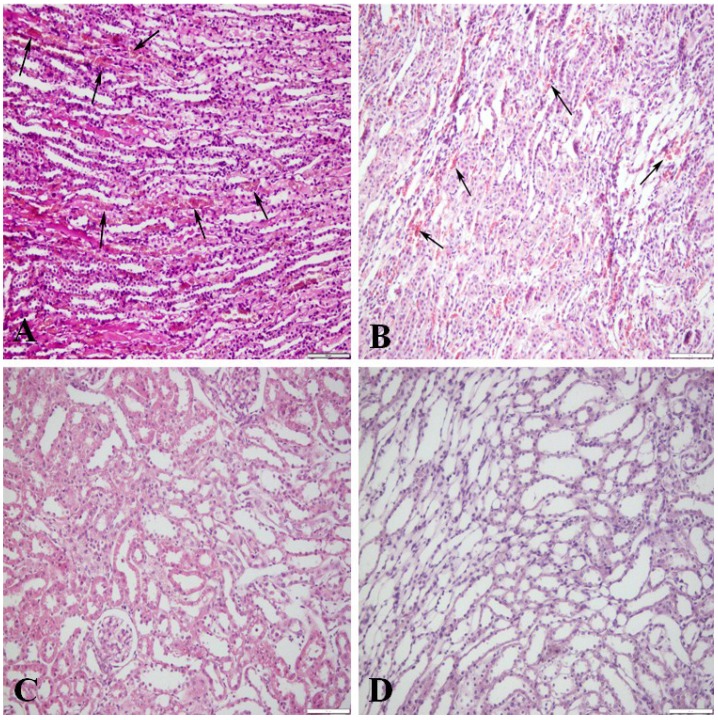
Kidney perfusion efficiency in rats receiving dietary ochratoxin A; contrasting sections stained with haematoxylin and eosin. (**A**) Not perfused; medullary blood vessels filled with erythrocytes (arrows); (**B**) Moderately efficient perfusion; numerous erythrocytes (arrows) in the medullary blood vessels; (**C**) Efficiently perfused kidney, no erythrocytes in the cortical blood vessels and glomeruli; (**D**) Efficiently perfused kidney; medullary blood vessels empty. Bar = 100 μm.

The study focuses on the situation within individuals and therefore tabulated findings (Table 1) show both individual data and gender group means where relevant. Generally, the measured OTA values in plasma were seven- to eight-fold higher than those in the normal (non-perfused) kidney. Qualitative monitoring of technical efficiency of flushing blood from the renal vasculature (Figure 1), assessed by histology of a small portion of each perfused kidney, clearly demonstrated the degree of success according to the reduction in the erythrocyte population that is naturally abundant in non-perfused kidney (Figure 1A). There was consistent correlation between the degree of perfusion efficiency predicted from histology (Figure 1B–D) and the percent efficiency values calculated from subsequent measurement of OTA in the perfused organs are described below (Table 1).

**Table 1:** Concentrations of OTA in paired perfused and non-perfused kidneys of male and female rats given dietary toxin, indicating perfusion efficiency and the estimated OTA content of kidneys according to the predicted size of the vascular compartment.

OTA in Plasma (µg/mL)	Estimated OTA in Blood (µg/mL)	Estimated OTA in Kidney at 25% Vascular Content (µg/g)	OTA in Normal Kidney (µg/g)	OTA in Perfused Kidney (µg/g)	Perfusion Efficiency (%)	Illustrated in Figure 1
Female						
18.1	10	2.5	3.2	0.27	90.5	C,D
11.8	6.5	1.6	1.8	1.16	37	
13.3	7.3	1.8	1.6	0.26	83.1	C,D
18.2	10	2.5	1.9	0.14	92.6	C,D
12.0	6.5	1.65	2.7	0.19	92.9	C,D
Mean ± SD	8.1 ± 1.8	2.01 ± 0.41	2.24 ± 0.61	0.40 ± 0.43	79.2 ± 23.9	
Male						
14.6	8.0	2.00	2.25	0.36	84.2	C,D
16.4	9.0	2.25	2.69	1.58	41.2	
21.2	11.6	2.91	2.63	1.31	50.3	B
17.2	9.5	2.36	3.02	1.22	59.7	B
23.6	13.0	3.24	1.89	0.08	95.9	C,D
14.8	8.2	2.04	2.25	0.03	98.4	C,D
Mean ± SD	9.9 ± 2.0	2.47 ± 0.50	2.46 ± 0.37	0.76 ± 0.68	71.6 ± 24.4	

In females, the plasma OTA concentration was in the range 12–18 μg/mL and kidney perfusions had generally been quite efficient (mean 79%). All perfused kidneys still contained a measurable amount of OTA, which is to be expected since perfusion could not be expected to be perfect. Additionally, some OTA must have remained within nephron epithelia, already destined for metabolism to ochratoxin alpha and subsequent excretion, and, therefore, was unaffected by vascular perfusion. Conversion of plasma OTA values to whole blood (assuming a standard 55% plasma component in blood [7]) before translation into an amount equivalent to that in kidney vasculature assuming a 25% compartment in kidney, gave a group mean value (2.01 ± 0.41 μg/mL) that quite closely matched the mean of the measured OTA value in kidneys (2.24 ± 0.61 μg/mL). These complementary findings support the conclusion that a large proportion of the OTA in kidney of these animals resided within the vascular compartment.

Similarly, in males, in which plasma OTA values (14–23 μg/mL) were a little higher than in the females, perfusion had also been quite efficient (mean 72%) and a little residual OTA was consistently detected after perfusion. OTA measured in whole kidneys (mean 2.46 μg/mL) was not significantly different from that in females. In addition, notably, the somewhat higher OTA content of feed given to the male rats did not disproportionately increase the measured OTA in either perfused or non-perfused kidneys. Application of a notional 25% vascular component for a kidney gave a remarkably close estimate of the non-perfused kidney content of OTA.

## 3. Discussion

Within the limitations of whole body perfusion technique and analytical efficiency for very small amounts of OTA in rat plasma and kidney, the present findings demonstrate that a very substantial proportion of the OTA in kidneys, when chronic OTA intake has reached a steady state in blood, is readily removable by vascular perfusion. Seven of the eleven present perfusions showed >80% difference between OTA content of pairs of perfused and non-perfused kidneys. That pharmacokinetic equilibrium would, from our experience, likely to be unilaterally carcinogenic for kidneys in a significant proportion of males after about nine months of continuous dietary exposure, slowly delivering ~30 mg of toxin [8,9]. A substantial proportion of this toxin will be excreted via kidney, with access to nephron epithelia by organic anion transporters and/or glomerular filtration. That currently seems more a matter of conjecture and has very little experimental demonstration. With a vast number of OTA molecules necessary to pass though a kidney to generate a lone cell in the OSOM with a significant mutation, it is probably impossible to track its origin. However, the recognition of putative pre-neoplastic nephron fragments by immunohistochemical histology [10] points to a source of early malignancy from which transformed cells could be obtained for molecular genetic analysis.

Application of the present study to several aspects of ochratoxicosis and associated publications can be made. A study in piglets, given dietary OTA for four weeks (118 ng/g in feed) [11], plasma OTA values at completion matched sympathetically with the OTA concentration in kidney and allowed the conclusion that reducing the OTA level in blood leads to its decrease in kidney. Applying the present principle, we note that if the pig renal vasculature was an ~28% component, it would have accounted for the whole OTA content of kidney.

In a very recent study [12], pubertal male Fischer rats (200 g) were given OTA-contaminated (10 µg/g) feed for six days in metabolism cages, twice the highest dose quoted in [13]. OTA had then reached 11 µg/mL in plasma and 1.44 µg/g in kidney, allowing a correlation calculated for the present context to equate to kidney OTA being exclusively in the vascular compartment if the latter had been only 23% of the organ. Further, OTA in brain, at a concentration of only 0.13 µg/g, pointed to the well-known and much smaller (~2%) vascular compartment of that organ. Nevertheless, authors clearly had great difficulty in explaining their findings, with reference to literature on mechanism, concluding that glomerular excretion of OTA was limited because of its high binding to plasma protein and that the high concentration in kidney is because it is a target for toxicity.

At first sight, inconsistency with the present findings is apparent in a gene-expression study [14], in which OTA concentration in plasma and kidney are defined purely as context for male rats gavaged daily (0.5 mg/kg b.w.) for up to three weeks. Applying the present principles to estimate kidney content only for a notional 25% vascular compartment *in vivo*, excluding any residence in renal parenchyma, reveals a 40%–45% shortfall. We attribute this partly to kidney harvest after loss of vascular turgor, while blood leaks due to capsule elasticity and partly due to volume losses during cutting, washing, and weighing tissue pieces before freezing in liquid nitrogen. Similarly, from the same laboratory [15], measurement of OTA content of kidney may not attain its full potential for estimating the proportion of OTA in renal parenchyma unless the vascular supply is first ligated under normal blood pressure, and blood allowed to clot *in situ*. However, this may not be compatible with a research objective. Notably, in a rat experiment [16], analogous to that in pigs from the same laboratory above [11], similar blood leakage and tissue freezing may explain the apparent shortfall in kidney OTA relative to that in plasma.

The present findings can also be applied to the OTA pharmacokinetic study [17] in which excised rat kidneys were uniquely flushed with saline. Since flushing of female kidney reduced renal OTA content by 90% from the ‘expected’ value, as by *in situ* perfusion in the present study, it is notable that the same technique in males achieved only a 42% reduction. For a single gavage dose on a body weight basis, and where males were much larger than females, we suggest that flushing of larger excised male kidneys via the renal artery may be less efficient. Therefore, perception of OTA “accumulation” in male kidneys in these circumstances, and implication for toxicology, may have been insecure.

Recent combined application of LC-MS analysis of ochratoxin A pharmacokinetics [18] performed acute and chronic experiments with Sprague-Dawley male rats. The acute dose 0.2 mg/kg b.w. was less than that in [17] (0.5 mg/kg b.w.) but was accompanied by a rather large ethanol component in the gavage vehicle. Correspondingly, the maximum plasma concentration (nearly 2 μg/mL) was less than in [17] (4.3 μg/mL), although achieved by 2 h and 4 h, respectively. Maximum concentration in several organs occurred at 4 h, in descending order kidney (400 ng/g, corresponding to nearly 2 μg/mL in plasma), lung, liver and heart, spleen, brain. By five days, OTA pharmacokinetics had stabilised, possibly as the ethanol influence subsided. In the new slower kinetics, measured OTA in plasma (~300 ng/mL) correlated closely with that in kidney (~45 ng/g) assuming a 25% vascular compartment.

In the chronic dosing experiment [18], organ analysis after 20 days of OTA exposure (0.1 mg/kg b.w.) gave an interesting rank order of OTA content, different from that after a single dose, namely lung, liver, heart and kidney, in descending order from 96 to 56 ng/g. Unfortunately, no plasma value is given and so authors missed a potential opportunity to deduce any connection(s) between organ OTA content and its vascularity. However, the rank change from kidney in top place after an acute dose is not surprising since lung is very highly vascular and has no excretory feature, whereas kidney provides an important and dynamic excretory and degradative function for OTA.

The present study also has wider implications. We similarly find the literature unclear concerning *in vivo* mechanism(s) in OTA transfer from renal vasculature to nephron epithelia. For example, a recent review [19] concludes that glomerular filtration is irrelevant because of the high degree of binding of OTA to plasma proteins. Therefore, the complex organic anion transfer (OAT) mechanism, on which much has been published particularly on *in vitro* studies in the last decade, is the way of renal excretion for OTA. However, we note that this trans-membranes concept requires transfer across both capillary wall and the basal membrane of nephrons and is energy-consuming, whereas escape of free OTA molecules through the semi-permeable fenestrations in the glomerular capillary cluster is easy, as it is for many small molecules for which crucial rescue and salvage mechanisms in renal proximal tubules sustain mammalian life. It is therefore impossible to ignore the glomerular excretion of OTA, and there is no experimental study to show the relative participation of any other excretory mechanism within kidney for this molecule.

Further concerning organic anion transmission, high affinity binding of OTA to plasma proteins is unlikely to be reversed while blood traverses peri-tubular cortical capillaries and glomeruli. Additionally notable, small plasma proteins (estimated size, 20 KDa) were recognised 30 years ago as binding OTA [20] and that magnitude of lipocalin molecule certainly passes through male rat glomerular filtration into urine [21], and could be involved in OTA excretion into the nephron lumen, bound to an α2u-globulin [22]. Ultimately, Anzai *et al.* [19] conclude that organic anion transporter function contributes to OTA accumulation in kidney. We have difficulty with this statement in view of the present findings, preferring to focus on mechanism of accumulation in blood, from which other apparent “accumulations” appear automatically in whole organ analysis when blood in vasculature is ignored.

The present equivalence of perfusion efficiency in both male and female rats does not obviously support the contention [1] that sex-hormone-dependent preferential uptake of OTA into proximal tubule cells via apical and basolateral organic anion transporters accounts for OTA accumulation in males as a key event in carcinogenicity. This is in a context, with selective use of literature, of citing the 2006 European Food Safety Authority opinion [23] that OTA has insufficient DNA reactivity for renal tumour formation, while ignoring the accessible description of an adduct structure already published [24].

Demonstration of the quantitative role of OTA as a vascular component extends logically, in kidney of rats exposed throughout adult life to OTA, to adducts with DNA that were recognised on 2D-chromatograms after ^32^P-postlabeling [25]. A few kidneys contained adenocarcinomas; adduct incidence throughout was of single or double digit amounts per 10^9^ nucleotides, demonstrating that OTA is a genotoxin. Subsequent attempts have been made to demonstrate contrarily a non-genotoxic tumourigenic mechanism for OTA [26,27], and there is conflicting interpretation of analytical methodology [28]. Several mechanisms have been studied, but even in [29], linked to a lifetime rat study, the gene-expression changes amongst four rats are difficult to link with carcinogenicity when there was only 25% tumour incidence. Subsequently, the model adduct C–C8 OTA 3'dGMP, formed semi-synthetically, was shown to co-chromatograph with the principal natural adduct derived from OTA-exposed rat kidney processed by ^32^P postlabeling [24]. Assuming, therefore, that OTA-DNA adducts do exist, albeit temporarily *in vivo*, it is important to know where they are and where incorrect repair might impose tumourigenic risk. This is important for understanding both experimental OTA-derived renal cancer in rats and also idiopathic human renal cancer that has never been attributed clearly to OTA. Detection of OTA-DNA adducts in blood has recently been reported for rats [30]. Since blood contains nucleated leukocytes they may reasonably contain adducts from circulating OTA; their contribution to kidney adducts could, therefore, be calculated to assess adducts in renal parenchyma.

Detection of DNA-OTA adducts in testis of mice given OTA [31] is not surprising, particularly concerning one co-chromatographing with C–C8 OTA 3'dGMP. Adducts in kidney and testis of new-borns, sourced via placental transfer from OTA-treated mothers; lower incidence in testis is consistent with its less vascularised structure. Indeed, were there any vascularised tissues without adducts? Nevertheless, limited evidence of exposure is hardly predictive of toxicological outcome at the short and relatively high artificial OTA exposure, especially since no direct connection between adducts and the initiation of OTA- derived experimental renal cancer is yet available. That OTA is “a biologically plausible cause of testicular cancer in man” [32] seems premature since authors omit to cite both the consistently-negative rat and mouse evidence (e.g., [33]) and the immunohistochemical differentiation of tumour in testis and kidney in experimental OTA oncology in rats [10].

The plant nephrotoxin aristolochic acid 1 forms four DNA adducts, in abundance more prominent than with OTA, but which appear to be much more persistent [34]. Blood seems not to have been studied, but could define their location in urinary tract parenchymal epithelia concerning experimental tumourigenesis and in humans, whether from misuse of herbal medicaments, or in traditional ethnobotanical usage or other undefined putative exposure in Balkan nephropathy hotspots. There is also the elusive silent nephropathic metabolite of the common food-borne mould *Penicillium polonicum*, causing rat renal apoptosis and one DNA adduct [35]. Chronic ingestion results in striking karyomegaly in the outer stripe of the outer medulla [36], a histopathological feature of topical interest also for OTA carcinogenicity [37].

In concluding that OTA measured in whole kidney may mostly be attributable to that in the vascular compartment, it follows that detection of DNA-OTA adducts in kidney, and in kidney tumours of humans and experimental animals, may also need careful re-interpretation, while still being a general indicator of exposure to the toxin. Assuming that, for example in male rats, adducts can be demonstrated specifically in nephron epithelia, a very low incidence of adduction, even below the current limit of detection, may be entirely sufficient so as, predictably via mis-repair, a single tumour might be initiated after many months of OTA insult. The obligatory long half-life characteristic of OTA in plasma [38] might provide the continuity of delivery to proximal tubule epithelia, even through intermittent dietary exposure, but this has not yet been tested

## 4. Materials and Methods

### 4.1. Animals, Housing Conditions and Experimental Procedure

Wistar rats (16 weeks old), from the Akdeniz University Animal Experiment Unit, were housed on sawdust bedding in groups of 4–5 in polycarbonate cages with stainless steel covers. Room temperature was maintained at 22 ± 2 °C with a relative humidity of 55% ± 10% and a day/night cycle of 12 h. Six males (average weight 300 g) and 5 females (average weight 210 g) were given experimental feed prepared by homogenizing standard rat diet with the same OTA-rich fermentation product (6 mg OTA g^−1^) as used previously [9] to provide 3 and 5 µg OTA g^−1^-contaminated feed for females and males, respectively. Rats were given 20 g feed/rat/day in stainless steel containers, and water *ad libitum.* The physical condition of each rat was assessed daily for any obvious clinical signs. The study was approved by Akdeniz University Local Animal Research Ethics Committee. The experiment continued for at least 2 months to achieve, from experience [13], an OTA concentration in circulating blood sufficient for meaningful measurements in plasma and kidneys but without any adverse clinical effect.

### 4.2. Kidney Perfusion

Perfusion was performed under physiological blood pressure (10–12 mm Hg) which was maintained by a peristaltic pump delivering physiological saline (50–60 mL) at 10–14 mL/min.

The rats were prepared for *in situ* perfusion of left kidney by anaesthetizing with sodium pentobarbital (50 mg/kg, intra peritoneal). After induction of anaesthesia, median laparotomy was performed for explosion of abdominal cavity and the right and left kidneys. The abdominal aorta was exposed by deflection of the intestines to the right side of the animal and dissected above and below the renal vessels. A loose tie was placed to the aorta above the renal arteries and below the superior mesenteric artery. For left kidney perfusion, a cannula was placed to the aorta above the genital arteries and tightly fixed in order to avoid any leakage of perfusion solution and consequent pressure lost.

Prior to left kidney perfusion, the right kidney artery and vein were tied together close to the kidney. The loose tie between the renal arteries and below the superior mesenteric artery was tied just before the perfusion solution was pumped to the aorta. Immediately after pumping the perfusion solution, the vein of the left kidney was cut close to the kidney for removal of the blood and perfusion solution.

Blood pressure of rats was measured with a pressure transducer connected to the cannula and a blood pressure recorder. The same system was also used for the measurement of perfusion pressure. Abdominal aorta blood pressure in the rats was found to vary between around 10–12 mm Hg. A continuous flow peristaltic pump was used for transfer of perfusion solution. To maintain the perfusion pressure within the above physiological blood pressure range, the flow rate was set to 10–14 mL/min. About 50–60 mL of lactated Ringer’s solution was used for perfusion of the kidney.

During perfusion, a change in colour of the left kidney, adjacent bowels and parenchyma was observed. Such changes were indicative of effective perfusion. Occasionally, technical problems such as displacement of the cannula from the aorta during perfusion or leakage of perfusion solution from aorta to upper part of body result in less efficient kidney perfusion by partial diversion of perfusate towards liver, lungs and brain. Consequently, less marked colour change during perfusion occurred in two out of six males and one out of five females, implying partial perfusion (Figure 1, Table 1). Nevertheless, all perfusions were included in the study.

After perfusion of the left kidney, both kidneys were removed and about a quarter of each kidney was put into 10% formaldehyde for histological analysis. The remaining three-quarters of each kidney was stored at −40 °C for OTA analysis.

### 4.3. OTA Extraction from Kidneys and Plasma

Frozen kidney (200–300 mg for non-perfused kidney and 350–450 mg for perfused kidney) was cut in small pieces and placed in 1 mL of 50 mM sodium phosphate buffer (pH 6.5) and and homogenized (Heildoph Silentcrusherm) for 1 min at 15,000 rpm. The homogeniser probe was washed between samples with water and rinsed with ethanol to prevent cross-contamination. Homogenates were kept at −40 °C for at least one day and then thawed at room temperature for 30 min. before OTA extraction. Ice-cold absolute ethanol (400 μL) and 20% trichloroacetic acid (50 μL) were added to 250 μL of tissue homogenate.

For plasma OTA extraction, 20 μL sample was added to 540 μL of extraction solution (20% trichloroacetic: ice-cold absolute ethanol, 1:8, *v*/*v*). The mixture was vortexed for 1 min and kept at room temperature for 15 min while mixing occasionally. The mixture was centrifuged at 9600 g for 10 min at 4 °C.

When necessary, the supernatant of organ or plasma extraction was diluted with (20% trichloroacetic acid: sodium phosphate buffer: absolute ethanol, 1:5:8 *v*/*v*) to obtain an OTA concentration in the range 1–50 ng mL^−1^. All samples were filtered using regenerated membrane filters (pore size 0.4 μm and disk size 4 mm, Corning) before HPLC injection.

### 4.4. OTA Analysis of Samples

OTA analyses were carried out in an accredited Turkish Research Council Food Quality Centre (TÜBİTAK-MAM, Gebze, Turkey) where residual mycotoxin analyses are performed on foods using validated methods. HPLC analyses were performed with a Shimadzu LC-20A liquid chromatographic system equipped with a fluorescence detector (RF-20A, Shimadzu HPLC, Kyoto, Japan). OTA was analysed on a 5 μm (15 cm × 0.4 cm) Tracer Extrasil ODS2 column (Thermo, Thermo Scientific, Waltham, MA, USA). The mobile phase was 29:29:42 (*v*/*v*) methanol-acetonitrile-sodium acetate (5 mM acidified to pH 2.6 with phosphoric acid); solvents were HPLC grade. The injection volume was 100 μL and flow rate was 1.5 mL/min. Chromatography was performed at 40 °C and monitored by fluorescence detection after optimising the excitation (333 nm) and emission (443 nm) wavelengths. Limits of detection (LOD) and quantification (LOQ) were 0.37 and 1 ng/mL, respectively, and OTA recovery for plasma and organs was 97% and 92%–96%, respectively.

### 4.5. Histological Examination for Perfusion Efficiency

Kidney samples (perfused and non-perfused) were collected during necropsy, fixed in 10% neutral buffered formalin and embedded in paraffin. Sections (5 μm) were stained with haematoxylin and eosin.

## 5. Conclusions

Analysis of perfused and non-perfused kidneys of rats with chronic, tolerable, circulating OTA from dietary exposure has demonstrated, for the first time, that most of the toxin resides in the well-defined vascular compartment. Consequently, focus should be on plasma protein-bound OTA, readily quantified, rather than on an artefact of apparent preferential accumulation in kidney. Recognition that most of the OTA in kidney *in vivo* can be in transit and toxicologically inert there emphasizes the need for better understanding of both whole body and organ pharmacokinetics of dietary OTA. Current neglect of the nucleate component of blood as a reservoir of nephrotoxin-DNA adducts, also in transit, may require reassessment of the significance of such adducts of, for example, OTA and aristolochic acid detected in healthy or malignant kidney.

## References

[1] Mally A. (2012). Ochratoxin A and mitotic disruption: Mode of action analysis of renal tumor formation by ochratoxin A. Toxicol. Sci..

[2] Hard G.C., Greig J.B., Castegnaro M., Chernozemsky I.N., Bartsch H. (1991). Comparative acute nephrotoxicity of *Penicillium aurantiogriseum* in rats and hamsters. Mycotoxins, Endemic Nephropathy and Urinary Tract Tumours.

[3] Cavanagh J.B., Holton J.L., Nolan C.C., Ray D.E., Naik J.T., Mantle P.G. (1998). The effects of the tremorgenic mycotoxin penitrem A on the rat cerebellum. Vet. Pathol..

[4] Mantle P.G., Nolan C.C. (2010). Pathological outcomes in kidney and brain in male Fischer rats given dietary ochratoxin A, commencing at one year of age. Toxins.

[5] Garcia-Sanz A., Rodriguez-Barbero A., Bentley M.D., Ritman E.L., Romero J.C. (1998). Three-dimensional microcomputed tomography of renal vasculature in rats. Hypertension.

[6] Bentley M.D., Jorgensen S.M., Lerman L.O., Ritman E.L., Romero J.C. (2007). Visualisation of three-dimensional nephron structure with microcomputed tomography. Anatom. Record.

[7] Blood Composition. http://sydney.edu.au/science/biology/learning/blood_composition.

[8] Mantle P.G., Dobrota M., Gillett C.E., Odell E.W., Pinder S.E. (2010). Oncological outcomes in rats given nephrocarcinogenic exposure to dietary ochratoxin A, followed by the tumour promoter sodium barbital for life: A pilot study. Toxins.

[9] Mantle P.G. (2009). Minimum tolerable exposure period and maximum threshold dietary intake of ochratoxin A for causing renal cancer in male Dark Agouti rats. Food Chem. Toxicol..

[10] Gazinska P., Herman D., Gillett C., Pinder S., Mantle P. (2012). Comparative immunohistochemical analysis of ochratoxin A tumourigenesis in rats and urinary tract carcinoma in humans; mechanistic significance of p-S6 ribosomal protein expression. Toxins.

[11] Aoudia N., Callu P., Grosjean F., Larondelle Y. (2009). Effectiveness of mycotoxin sequestration activity of micronized wheat fibres on distribution of ochratoxin A in plasma, liver and kidney of piglets fed a naturally contaminated diet. Food Chem. Toxicol..

[12] Abbas Z., Blank R., Wein S., Wolffram S. (2013). Effect of quercitin on the toxicokinetics of ochratoxin A in rats. Food Add. Contam..

[13] Mantle P.G. (2008). Interpretation of the pharmacokinetics of ochratoxin A in the blood plasma of rats during and after acute or chronic ingestion. Food Chem. Toxicol..

[14] Arbillaga L., Vettorazzi A., Gil A.G., van Delft J.H.M., Garcia-Jalon J.A., Lopez de Cerain A. (2008). Gene expression changes induced by ochratoxin A in renal and hepatic tissues of male F344 rat after oral repeated administration. Toxicol. Appl. Pharmacol..

[15] Corcuera L.A., Vettorazzi A., Arbillaga L., Gonzales-Penas E., Lopez de Cerain A. (2012). An approach to the toxicity and toxicokinetics of aflatoxin B1 and ochratoxin A alter simultaneous oral administration to fasted F344 rats. Food Chem. Toxicol..

[16] Aoudia N., Tangni E.K., Larondelle Y. (2008). Distribution of ochratoxin A in plasma and tissues of rats fed a naturally contaminated diet amended with micronized wheat fibres: Effectiveness of mycotoxin sequestering activity. Food Chem. Toxicol..

[17] Zepnik H., Volkel W., Dekant W. (2003). Toxicokinetics of the mycotoxin ochratoxin A in F 344 rats after oral administration. Toxicol. Appl. Pharmacol..

[18] Han Z., Zhao Z., Shi J., Liao Y., Zhao Z., Zhang D., Wu Y., Saeger S., Wu A. (2013). Combinatorial approach of LC-MS/MS and LC-TOF-MS for uncovering *in vivo* kinetics and biotransformation of ochratoxin A in the rat. J. Chromatog. B.

[19] Anzai N., Jutabha P., Endon H. (2010). Molecular mechanisms of ochratoxin A transport in the kidney. Toxins.

[20] Stojkovic R., Hult K., Gamulin S., Plestina R. (1984). High affinity binding of ochratoxin A to plasma constituents. Biochem. Int..

[21] Vettorazzi A., Wait R., Nagy J., Monreal J.L., Mantle P. (2013). Changes in male rat urinary protein profile during puberty: A pilot study. BMC Res. Notes.

[22] Mantle P.G., Nagy J.M. (2008). Binding of ochratoxin A to a urinary globulin: A new concept to account for gender difference in rat nephrocarcinogenic responses. Int. J. Mol. Sci..

[23] EFSA (2006). Opinion of the scientific panel on contaminants in the food chain on a request from the Commission related to ochratoxin A in food. EFSA J..

[24] Mantle P.G., Faucet-Marquis V., Manderville R.A., Squillaci B., Pfohl-Leszkowicz A. (2010). Structures of covalent adducts between DNA and ochratoxin A: A new factor in debate about genotoxicity and human risk assessment. Chem. Res. Toxicol..

[25] Castegnaro M., Mohr U., Pfohl-Leszkowicz A., Esteve J., Steinmann J., Tillmann T., Michelon J., Bartsch H. (1998). Sex- and strain specific induction of renal tumours by ochratoxin A in rats correlates with DNA adduction. Int. J. Cancer.

[26] Mally A., Zepnik H., Wanek P., Eder E., Dingley K., Ihmels H., Volkel W., Dekant W. (2004). Ochratoxin A: Lack of formation of covalent DNA adducts. Chem. Res. Toxicol..

[27] Turesky R.J. (2005). Perspective: Ochratoxin A is not a genotoxic carcinogen. Chem. Res. Toxicol..

[28] Delatour T., Mally A., Dekant W., Marin-Kuan M., Schilter B., Cavin C. (2009). Answer to Prof. Pfohl-Leszkowicz’s letter to Editor. Mol. Nutr. Food Res..

[29] Marin-Kuan M., Nestler S., Verguet C., Bezencon C., Piguet D., Mansourian R., Holzwarth J., Grigorov M., Delatour T., Mantle P. (2006). A toxicogenomics approach to identify new plausible epigenetic mechanisms of ochratoxin A carcinogenicity in rat. Toxicol. Sci..

[30] Pfohl-Leszkowicz A., Faucet-Marquis V., Tozlovanu M., Peraica M., Stefanovic V., Manderville R. C8-2'-Deoxyguanosine ochratoxin A-adducts and OTA metabolites in biologic fluids as biomarkers of OTA exposure. Proceedings of the MycoRed International Conference.

[31] Jennings-Gee J.E., Tozlovanu M., Manderville R., Miller M.S., Pfohl-Leszkowicz A., Schwartz G.G. (2010). Ochratoxin A: In utero exposure in mice induces adducts in testicular DNA. Toxins.

[32] Malir F., Ostry V., Pfohl-Leszkowicz A., Novotna E. (2013). Ochratoxin A: Development and reproductive toxicity—An overview. Birth Defects Res..

[33] Boorman G.A. (1989). Toxicology and Carcinogenesis Studies of Ochratoxin A.

[34] Schmeiser H.H., Nortier J.L., Singh R., da Costa G.G., Sennesael J., Cassuto-Viguier E., Ambrosetti D., Rorive S., Pozdzik A., Phillips D.H., Stiborova M. (2014). Exceptionally long-term persistence of DNA adducts formed by carcinogenic aristolochic acid 1 in renal tissue from patients with aristolochic acid nephropathy. Int. J. Cancer.

[35] Miljkovic A., Pfohl-Leszkowicz A., Dobrota M., Mantle P.G. (2003). Comparative responses to mode of oral administration and dose of ochratoxin A or nephrotoxic extract of *Penicillium polonicum* in rats. Exp. Toxic. Pathol..

[36] Mantle P.G., McHugh K.M., Fincham J.E. (2010). Contrasting nephropathic responses to oral administration of extract of cultured *Penicillium polonicum* in rat and primate. Toxins.

[37] Taniae E., Yafune A., Nakajima M., Hayashi S.-M., Nakane F., Itahashi M., Shibutani M. (2014). Ochratoxin A induced karyomegaly and cell cycle aberrations in renal tubular calls without relation to induction of oxidative stress. Toxicol. Lett..

[38] Hagelberg S., Hult K., Fuchs R. (1989). Toxicokinetics of ochratoxin A in several species and its plasma-binding properties. J. Appl. Toxicol..

